# Modified Plastic Optical Fibers Combined with Molecularly Imprinted Polymers and Gold Nanorods for Furfural Detection at the Picomolar Level via Plasmonic Phenomena

**DOI:** 10.3390/polym18111413

**Published:** 2026-06-05

**Authors:** Rosalba Pitruzzella, Dalila Cicatiello, Chiara Marzano, Luca Pasquale Renzullo, Viktor Zabolotnii, Roman Viter, Luigi Zeni, Maria Pesavento, Giancarla Alberti, Nunzio Cennamo

**Affiliations:** 1 Department of Women, Child and General and Specialized Surgery, University of Campania Luigi Vanvitelli, Via Luigi de Crecchio 2, 80138 Naples, Italy; rosalba.pitruzzella@unicampania.it (R.P.); 2 Department of Engineering, University of Campania Luigi Vanvitelli, Via Roma 29, 81031 Aversa, Italy; dalila.cicatiello@studenti.unicampania.it (D.C.); chiara.marzano@unicampania.it (C.M.); lucapasquale.renzullo@unicampania.it (L.P.R.); luigi.zeni@unicampania.it (L.Z.); 3 Faculty of Science and Technology, University of Latvia, 19 Raina Blvd, LV 1586 Riga, Latvia; viktor.zabolotnii@lu.lv (V.Z.); roman.viter@lu.lv (R.V.); 4 Department of Chemistry, University of Pavia, Via Taramelli 12, 27100 Pavia, Italy; maria.pesavento@optosensing.it (M.P.)

**Keywords:** plastic optical fibers (POFs), localized surface plasmon resonance (LSPR), molecularly imprinted polymers (MIPs), optical-chemical sensor, 2-furaldehyde (2-FAL), gold nanorods (GNRs)

## Abstract

This work presents an intrinsic optical fiber sensor based on plasmonic phenomena in modified plastic optical fibers (POFs). The sensing area is achieved by replacing the polymethyl methacrylate (PMMA) core with a molecularly imprinted polymer (MIP) containing gold nanorods (GNRs). Thus, in the sensing area, the MIP acts as both a selective recognition element and an optically sensitive guiding medium where plasmonic phenomena occur. This optical–chemical configuration has been developed as a proof-of-concept for the detection of furfural in aqueous solution. The proposed sensor achieves a limit of detection (LOD) of 27 pM, demonstrates high selectivity for the analyte of interest, and is applicable even in real-world scenarios, as demonstrated by experimental results (a commercially available infant milk). The proposed sensor presents a significant enhancement of the sensor response, of about six orders of magnitude, compared to a conventional configuration where the same (or a similar) mixture of MIP/GNRs is spun over the exposed PMMA of a D-shaped POF area for comparison. Notably, even if this study has been carried out via a proof-of-concept in furfural detection, this substantial improvement is achieved while preserving a simple, portable, and cost-effective optical setup, highlighting the potential of this sensing strategy for the development of highly selective sensors by changing the MIP template.

## 1. Introduction

Plastic optical fibers (POFs) have attracted considerable interest in recent years due to their advantageous properties, including high flexibility, wide strain limits, ease of handling, and high numerical aperture [1,2]. These characteristics make POF particularly well-suited for the development of simple, highly sensitive, and cost-effective sensing systems [3].

Thanks to these unique characteristics, POF-based sensors have been successfully applied across a wide range of fields, including industrial monitoring [4,5], healthcare [6,7], structural health monitoring [8,9], food and beverage safety [10,11]. In these applications, POFs typically act as sensing waveguides, enabling efficient light propagation and interaction with the surrounding environment. Furthermore, these systems are particularly attractive for remote sensing applications, where optical fibers enable the detection of hazardous substances while keeping the operator at a safe distance [12].

POF-based sensors can generally be classified into intrinsic and extrinsic configurations. In intrinsic sensors, the fiber interacts directly with the medium under investigation, often exploiting the POF’s multimodal waveguide characteristics [13]. In contrast, extrinsic sensors use external sensing elements, leveraging the POF’s high numerical aperture to efficiently collect and transmit light to and from the sensing region [14].

Several POF-based detection approaches rely on plasmonic phenomena, particularly surface plasmon resonance (SPR) and localized surface plasmon resonance (LSPR). SPR is a sensitive technique for detecting small changes in the refractive index (RI) at the interface between a continuous metallic layer and a dielectric medium [15,16]. On the other hand, LSPR is excited by metal nanoparticles or nanostructured surfaces, thereby enhancing the local electromagnetic field and improving binding sensitivity [17,18].

To achieve high selectivity toward specific target analytes, SPR- and LSPR-based sensors are often combined with molecular recognition elements (MREs), which enable selective binding of the target analyte. These MREs can be biological, such as proteins, nucleic acids, enzymes, antibodies, and aptamers, or biomimetic and synthetic, such as molecularly imprinted polymers (MIPs) [19].

Compared to biological receptors, MIPs offer several advantages, including greater chemical and physical stability outside natural environments, high reproducibility, lower production costs, and the ability to operate in harsh conditions over a wide range of pH and temperature [20]. These properties make MIPs particularly promising for the development of robust, reliable optical selective sensors for real-world applications.

For instance, Sharma et al. [21] developed a highly sensitive optical chemical sensor based on an optical fiber (OF) and SPR for ethanol detection, using a D-shaped OF as a waveguide. Nanolayers of gold and graphene are applied to induce SPR and enhance the sensor’s performance, while an MIP coating increases selectivity and interaction with ethanol molecules. The proposed sensor shows great potential for real-time alcohol detection and for applications in food and beverage safety monitoring [21].

Meanwhile, Cennamo et al. have proposed a POF-based optical chemical sensor that exploits LSPR to selectively detect 2,4,6-trinitrotoluene (TNT). In this configuration, the LSPR is excited via five-branch gold nanostars (GNS) embedded in an MIP matrix specifically designed for TNT recognition, providing a promising strategy for the development of highly selective and sensitive optical detection systems for explosive compounds [22].

Another advantage of MIPs as biomimetic receptors is their potential for direct integration into optical configurations. Specifically, MIPs can be employed not only as a functional overlayer grafted onto the sensitive region, but also as the waveguide itself, enabling a more compact and intrinsically sensitive sensing architecture [23].

For instance, Arcadio et al. [23] presented an innovative optical chemical sensing approach in which a MIP for 2-furaldehyde (2-FAL) was used as the core of an optical waveguide. More specifically, a channel was fabricated within a substrate using a computer numerical control (CNC) machine, and the optical–chemical platform was interrogated with two POFs integrated directly into the system [23].

This study presents the development of modified POFs combined with MIPs for the selective detection of 2-FAL as the target analyte. In particular, 2-FAL was selected as a proof-of-concept due to extensive support in the literature; it is a compound formed by the thermal degradation of sugars [24] and, at high concentrations, is harmful to humans [25]. In power transformers, it represents a key indicator of the aging of cellulose-based insulating paper, and high levels are associated with an increased risk of failure [26].

In this work, as a first step, a previously developed MIP-GNS sensor based on a POF platform [22] was re-examined. In this case, the TNT-selective MIP was replaced with a 2-FAL-selective MIP, while maintaining a similar mixture composition. Furthermore, the GNSs were replaced with alternative gold nanostructures, specifically gold nanorods (GNRs) [27] (GNRs were used instead of GNSs). This configuration, named “conventional D-shaped POF coated with MIP-GNRs,” has been developed and tested in order to compare the performance of the proposed optical chemical sensor. As a second step, the work presents the kernel of the manuscript: the development of an optical–chemical sensor configuration named “Trench in POF filled by MIP-GNRs”. In particular, the proposed sensor configuration is based on a trench etched into the POF, which is filled with a mixture of MIP and GNRs, and then polymerized within the trench, forming the sensitive waveguide core. This represents the first example of an intrinsic POF sensor configuration that exploits MIP-GNRs as the optical waveguide core, replacing PMMA to improve the sensor’s chemical performance.

Furthermore, the selectivity of the proposed trench-based MIP-GNRs sensor configuration was evaluated by testing the sensor with potential interfering substances and comparing the results with those obtained from a non-imprinted polymer (NIP) sensor. Moreover, to demonstrate its real-world applicability, the proposed sensor was successfully used to detect 2-FAL in a complex matrix, specifically infant liquid milk. Finally, the performance of this detection strategy was compared with that of state-of-the-art LSPR- and MIP-based sensors, highlighting its superior sensitivity and the advantages of integrating MIPs directly into the optical waveguide.

## 2. Materials and Methods

### 2.1. Reagents–Chemicals

All reagents, including 2-furaldehyde (2-FAL), methacrylic acid (MAA), divinylbenzene (DVB), 2,2′-azobisisobutyronitrile (AIBN), Atrazine (ATZ) and 5-Hydroxymethylfurfural (5-HMF), were of analytical grade and purchased from Merck KGaA, Darmstadt, Germany.

For the synthesis of GNRs ammonium metatungstate hydrate (AMT, Merck KGaA, Darmstadt, Germany), Hydrogen tetrachloroaurate(III) hydrate 99.9% (Alfa Aesar, Thermo Fisher Scientific, Waltham, MA, USA), Polyvinylpyrrolidone (PVP, Merck KGaA, Darmstadt, Germany) (Mw Mw 1,300,000), Polyacrylonitrile (PAN, Merck KGaA, Darmstadt, Germany), N, N-Dimethylformamide (DMF, Merck KGaA, Darmstadt, Germany) were used without any specific preparations.

### 2.2. Electrospinning for Nanorod Fabrications

GNRs have been produced via a core-shell electrospinning method, as previously reported [27,28]. Solutions for electrospinning were prepared as follows:

Core solution: 0.41 g of PAN was added to preheated (85 °C) 5 mL of DMF and stirred at 200 rpm until dissolution. Subsequently, 0.35 g of AMT was added to the solution, and the mixture was stirred overnight at room temperature.

Shell solution: 1 g of PVP added to preheated (50 °C) 5 mL of DMF and stirred at 200 rpm until dissolution. Then, 0.057 g of HAuCl_4_ was added to the solution, and the mixture was stirred overnight at room temperature.

Both solutions were loaded into 5 mL plastic syringes, attached to a coaxial needle (Linari Engineering, Pisa, Italy) with plastic tubes and placed in separate syringe pumps. Pump speeds were set to 300 μL/h for the core solution and 400 μL/h for the shell solution. The needle has an inner diameter of 0.5 mm, an outer diameter of 1 mm, and has been installed in the spinning chamber 20 cm above the collector. The collector was covered by aluminum foil. The collector’s rotation speed was set to 200 rpm. Between the needle and the collector, a 20 kV potential was applied.

The received fibers were dried in a vacuum overnight and annealed in the furnace at 500 °C for 1 h.

### 2.3. Characterization of Structure and Optical Properties of Core-Shell Nanofibers

X-ray diffraction (Bruker D8 diffractometer, CuKα radiation, Karlsruhe, Germany) has been used to identify the phases of the developed core-shell nanofibers. The structural properties of the deposited nanofibers were investigated using SEM (Hitachi, Tokyo, Japan).

Optical properties of the core-shell nanofibers have been studied by diffuse reflectance spectroscopy in the UV–Visible range. Ocean Optics fiber optic light source (DH2000, 250–900 nm, Orlando, FL, USA), integrating sphere (Ocean Optics, IS-8, Orlando, FL, USA) and fiber optic spectrometer (Ocean Optics HR4000, Orlando, FL, USA).

### 2.4. Preparation of MIP and NIP Mixture

The MIP was prepared by a non-covalent imprinting method, using a simple radical polymerization procedure [23]. A prepolymeric mixture was formulated with a molar ratio of 2-FAL:MAA:DVB equal to 1:4:40. Initially, MAA was dispersed in DVB via sonication. Subsequently, 2-FAL was introduced as the template, and the solution was deaerated under a nitrogen flow for 10 min. An excess amount of AIBN was then added to the mixture.

DVB was used in large amounts because it serves as both a crosslinking agent and a solvent, yielding a rigid polymer. At this stage, the nanostructures (GNRs) synthesized as described in Section 2.2 were dispersed in the prepolymeric mixture and subsequently used to fabricate the two sensor configurations described in Section 3.1 and Section 3.2. The resulting liquid mixture was deposited into the channel and then thermally polymerized in an oven at 75 °C overnight.

The non-imprinted polymer (NIP) was prepared using the same procedure, except that the analyte was omitted from the prepolymeric mixture.

### 2.5. Real Sample

To evaluate the applicability of the proposed sensor in real matrices, a commercially available infant milk (BBmilk 0–12 BIO, produced by Buona SpA Società Benefit, Sesto Fiorentino, Italy) was selected. The product is an organic (biologically sourced) ready-to-use formula, sterilized via UHT treatment and packaged in a 500 mL Tetra Pak container. The sample was manufactured and packaged at the facility in Ozzano Taro (PR), Italy. The matrix consists of a complex mixture of proteins, lipids, carbohydrates (primarily lactose), vitamins, and mineral salts.

### 2.6. Experimental Setup

**Figure 1:**
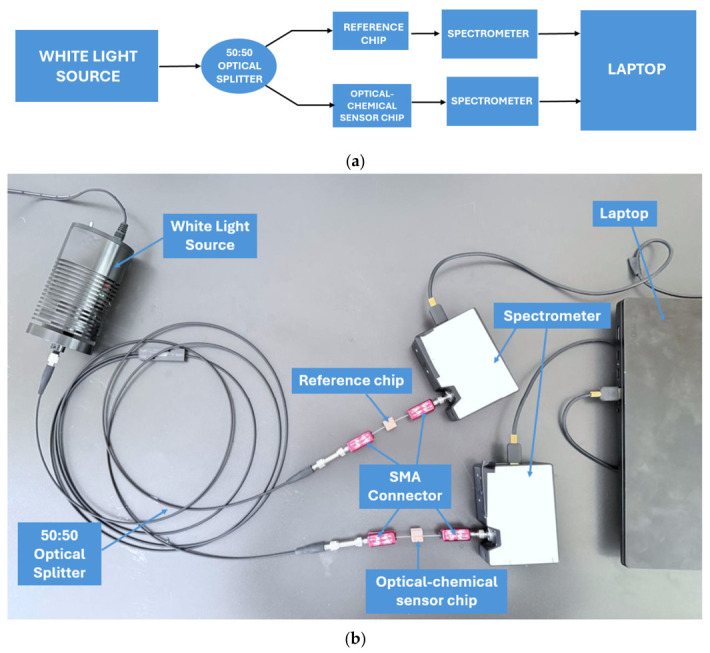
(**a**) Schematic diagram of the sensor system and (**b**) picture of the experimental setup used to test the optical–chemical sensor chips.

For both configurations, the experimental setup was designed to measure the spectrum of transmitted light. It consists of a white light source, represented by a halogen lamp (HL2000-LL, purchased from Ocean Optics, Orlando, FL, USA), which has a broad emission range between 360 nm and 1700 nm, ensuring wide spectral coverage; two spectrometers (SR-6VN500, purchased from Ocean Optics, Orlando, FL, USA), operating in the wavelength range between 200 and 850 nm; and a 50:50 optical splitter. All connections were made via SMA connectors. Figure 1a shows a schematic diagram of the proposed sensor system, and Figure 1b shows a picture of the experimental setup.

Specifically, the halogen lamp was connected via an optical splitter to the optical chemical sensor chip on one side and to the reference chip on the other. As a reference chip, an optical platform combined with NIP, but without GNRs, was used. The data (transmitted light from both chips) were acquired by the spectrometers and transmitted to a laptop, where they were analyzed using dedicated software from Ocean Optics.

More specifically, the reference chip presents an optical path similar to that of the sensor chip, without plasmonic phenomena or specific binding sites; therefore, it can serve as a reference transmitted spectrum for each tested sample to normalize the sensor chip’s transmitted spectra. The experimental setup used is characterized by simplicity, portability, and affordability, making it particularly suitable for applications requiring rapid, easy, and low-cost analysis.

### 2.7. Measurement and Data Analysis

In both configurations, all measurements were performed according to the same experimental protocol. Specifically, 70 μL of aqueous solutions containing different concentrations of the target molecule (2-FAL) were dropped onto both the sensor and reference surfaces, without the need for complex microfluidics, and incubated at room temperature for 10 min to promote interaction between the receptor and the analyte in the MIP region.

Between incubations, both the sensor and reference surfaces were thoroughly rinsed several times with ultrapure water to prevent surface contamination while maintaining the polymer layer’s hydration. After each incubation and washing step, transmitted spectra were recorded using ultrapure water as the bulk solution on both sensor and reference surfaces.

The experimentally obtained transmitted spectra of the sensors were normalized with respect to the reference spectrum of an NIP-based chip without GNRs, which has a similar optical path, in order to obtain the LSPR spectra. This normalization procedure minimizes potential temperature drift, environmental and bulk-solution contributions to the sensor response.

The normalization process was performed using MATLAB software (version R2022b, MathWorks, Natick, MA, USA); the LSPR spectra were smoothed using MATLAB’s “smooth” function (smoothing factor of 100) and subsequently shifted by a pure translation along the *y*-axis to better compare the minimum values of the LSPR spectra.

By adopting this protocol, only the resonance wavelength shift associated with the specific binding between the receptor and the analyte was measured, effectively minimizing contributions arising from non-specific interactions.

The dose–response curve is modeled by the Langmuir equation (Equation (1)).

(1) $$\Delta \lambda_{c}=\lambda_{c}-\lambda_{0}=\Delta \lambda_{max}\cdot \left(\frac{c}{K+c}\right)$$

where *λ*_*c*_ is the resonance wavelength at the 2-FAL concentration (c), *λ*_0_ is the resonance wavelength of the blank, Δ*λ*_max_ is the resonance wavelength variation at the saturation concentration, and K is the dissociation constant. Δ*λ*_max_ and K were obtained from a non-linear fitting of the experimental data using OriginPro software 2015 (32-bit) Srl b9.2.257, OriginLab Corp., Northampton, MA, USA.

When *c* is much smaller than K, Equation (1) can be approximated by a linear equation $\Delta \lambda_{c}=\Delta \lambda_{max}\cdot \left(\frac{c}{k}\right)$, allowing the calculation of the sensitivity at low concentrations (S_low c_), i.e., the slope $\frac{\Delta \lambda_{max}}{K}$ of the straight line; the parameter K is the reciprocal of the target molecule’s affinity constant (*K_aff_*) for the recognition site of the MIP.

The error bar on the experimental points in the dose–response curves correspond to the maximum standard deviation observed across the three sensors (*n* = 3) under the same experimental conditions. The experimentally obtained standard deviation of the blank solution across measurement replicates was not used to estimate the sensor performance parameters.

It is important to underline that the experimentally obtained standard deviation value was not used to estimate the analytical parameters. In fact, to estimate the detection limit (LOD), the model error (the standard deviation of the blank obtained from Langmuir fitting parameters) is used.

Therefore, the experimentally maximum standard deviation is used to evaluate the model error. In particular, the LOD can be approximated as 3.3 times the standard deviation of the intercept divided by S_low c_.

## 3. Optical Chemical Sensors

### 3.1. A Conventional D-Shaped POF Covered by MIP-GNRs Layer: Fabrication Process

The fabrication process of the proposed sensing configuration, named “Conventional D-shaped POF covered by MIP-GNRs”, was based on a previously reported approach [22]. However, in the present work, modifications relative to [22] were introduced, including the nanostructures employed (gold nanorods instead of nanostars), and the prepolymeric mixture (tailored for 2-FAL detection rather than TNT).

Briefly, the conventional D-shaped POF covered with MIP-GNRs consists of a D-shaped POF coated with a sensitive layer, i.e., a MIP layer embedding GNRs. More specifically, the optical platform was fabricated by polishing a POF to remove the cladding and a portion of the core, resulting in a D-shaped cross-section. The POF, featuring a PMMA core (980 µm in diameter, RI = 1.49) and a fluorinated polymer cladding (20 µm in diameter, RI = 1.41), was embedded in a resin support block to ensure mechanical stability and facilitate polishing. The exposed core region was obtained by a two-step polishing process using progressively finer abrasive papers (first 5 µm grit, then 1 µm grit) [29]. The sensitive area of the sensor is 10 mm long and approximately 700 µm wide.

The nanostructures obtained as explained in Section 2.2 are weighed, and 0.5 mg are dispersed in 1 mL of the 2-FAL-specific MIP prepolymer mixture (about RI = 1.61) [30] prepared as explained in Section 2.4. They are mixed using ultrasound until completely dissolved, then vortexed before use. Subsequently, 100 μL of the resulting mixture was deposited on the sensor’s sensitive area by spinning for 2 min at 1000 rpm to achieve an ultra-thin layer. Thermal polymerization of the mixture was carried out under controlled conditions in an oven at 75 °C for 12 h. After the polymerization step, the template is removed by repeated washing with water and ethanol, thereby emptying the recognition sites, which are now ready to host the target analyte. As a result, the sensitive layer was ultra-thin and, despite its high refractive index, acted as a non-guiding layer. Together, a NIP-based reference chip was prepared using the same protocol, but without the template molecule and without gold nanostructures. This NIP-based sensor serves only as a reference to normalize the optical signal, ensuring that the detected optical response is attributed specifically to the interaction between the MIP’s sites and the analyte.

### 3.2. Trench in POF Filled by MIP-GNRs: Fabrication Process

**Figure 2:**
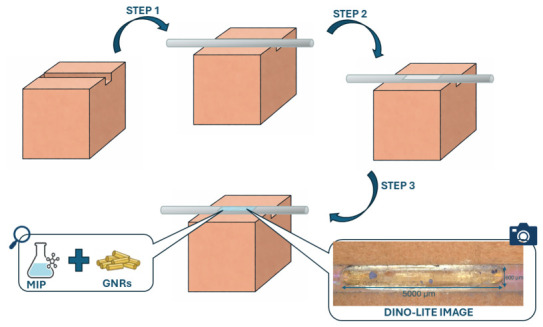
Fabrication process outline of the “Trench in POF filled by MIP-GNRs”. The zoom-in shows the trench filled with MIP-GNR with the corresponding measurements, captured using the Dino-Lite digital microscope (AnMo Electronics Corporation, New Taipei City, Taiwan).

The fabrication steps for the “Trench in POF filled by MIP-GNRs” sensor are schematically illustrated in Figure 2. Specifically, the same POF embedded in the support resin block used in the “Conventional D-shaped POF covered by MIP-GNRs” was employed. In this configuration, a micro-trench was machined in the POF using a CNC machine. The dimensions of the micro-trench are: 5000 µm in length, 600 µm in width, and 300 µm in depth. The micro-trench was then completely filled with the MIP prepolymer mixture for 2-FAL and GNRs, prepared in the same manner as for the previous sensor configuration, as described in Section 3.1. Thermal polymerization of the mixture was carried out in an oven at 75 °C for 12 h under controlled conditions.

After polymerization, the template was removed by repeated washing with water and ethanol, generating selective recognition sites complementary to the target analyte.

In this sensor configuration, the roughness of the realized trench promotes scattering, enabling light propagation within the MIP-GNR region of the POF.

Similarly, a reference chip was fabricated; in this case, the microchannel was filled with an NIP mixture free of GNRs. Similar to the D-shaped configuration described in Section 3.1, this NIP-based chip was used only as a reference to normalize the signal, thereby accounting for nonspecific variations and ensuring the accuracy of the optical sensor response.

In this optical–chemical sensor configuration, the MIP is not simply an overlying layer grafted onto the sensing area, but constitutes the core of the waveguide. When 2-FAL (analyte) binds to the selective recognition sites of MIP, two distinct phenomena occur: (i) the dielectric RI surrounding the GNRs locally changes; (ii) a significant change is observed in the guiding properties (in terms of transmitted light) of the proposed sensitive optical waveguide as a result of the change in the RI of the waveguide core (i.e., the MIP). The coexistence of these two phenomena enhances binding, inducing a significant and measurable shift in the LSPR spectral response (typically observed as a shift in the resonance wavelength) that is directly correlated with the analyte concentration [22,31].

## 4. Results

### 4.1. GNRs Characterization

**Figure 3:**
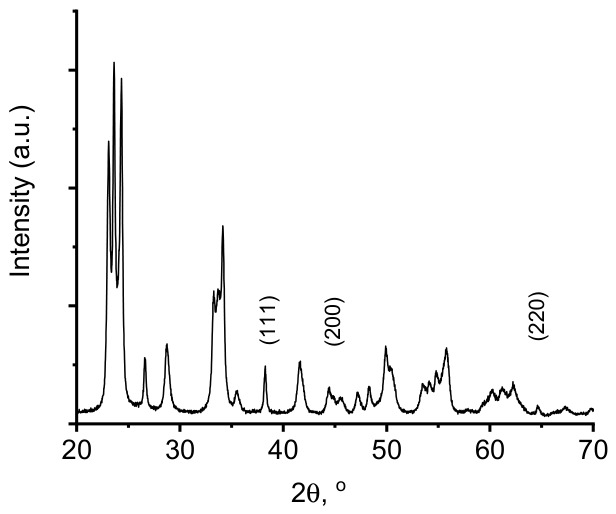
XRD of WO_3_/Au nanofibers.

XRD spectra of the WO_3_/Au nanofibers are shown in Figure 3. Analysis of the XRD spectra showed WO_3_ peaks, located at 2θ°: 23.1, 23.62, 24, 24.34, 26.61, 28.71, 33.26, 33.71, 34.12, 35.51, 41.6, 45.56, 47.2, 48.29, 49.88, 50.36, 53.45, 54.1, 54.7, 55.7, 57.7, 59.41, 60.18, 61.13, 62.21, 66.28, 67.42. Based on XRD data analysis, the fabricated WO_3_ is mainly presented by a mixture of orthorhombic (JCPDS card 20-1324) and monoclinic (JCPDS card 83-0950) phases. The XRD peaks at 2θ°, 38.21, 44.43 and 64.65, correspond to Au nanostructures (JCPDS card 04-0784).

**Figure 4:**
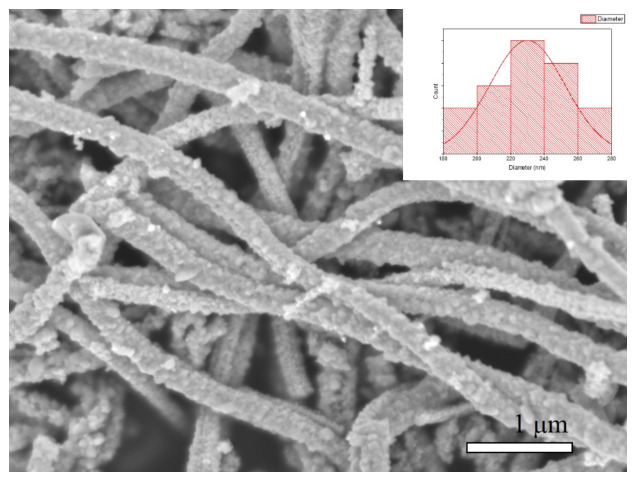
SEM imaging and diameter distribution of WO_3_/Au nanofibers.

SEM images of the WO_3_/Au nanofibers are presented in Figure 4, respectively. The SEM images show well-shaped nanofibers fabricated by electrospinning. The dimensions of the fibers were 232 ± 41 nm with an average length of the nanofibers of 12 μm.

**Figure 5:**
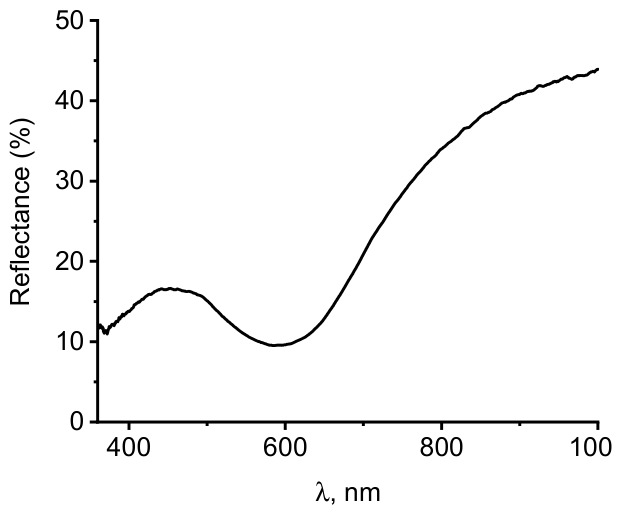
Diffuse reflectance spectra of WO_3_/Au nanofibers in air.

Optical properties of the WO_3_/Au nanofibers have been investigated by diffuse reflectance spectroscopy in air (Figure 5). The WO_3_/Au nanofibers had two specific absorption ranges from 360 to 420 nm and from 550 to 800 nm. The first UV part corresponds to absorption edge of WO_3_ [32]. Reflectance minimum, centered at 588 nm, corresponds to LSPR absorption by Au nanostructures [33]. In the characterization reported in Figure 5, the nanostructures (WO_3_/Au nanofibers) were pressed into a specific cylindrical form with a 40 mm diameter and a 0.5 mm thickness. Under these conditions, the transmittance of this system is 0% as all light will be scattered, absorbed or reflected. Conversely, in the proposed optical fiber sensor configurations, the gold nanostructures (WO_3_/Au nanofibers) are dispersed within the MIP matrix. Therefore, WO_3_/Au nanofibers are surrounded by a medium with a refractive index significantly higher than that of air or water.

### 4.2. Conventional D-Shaped POF Covered by MIP-GNRs: Dose–Response Curve for 2-FAL Detection in Ultrapure Water

**Figure 6:**
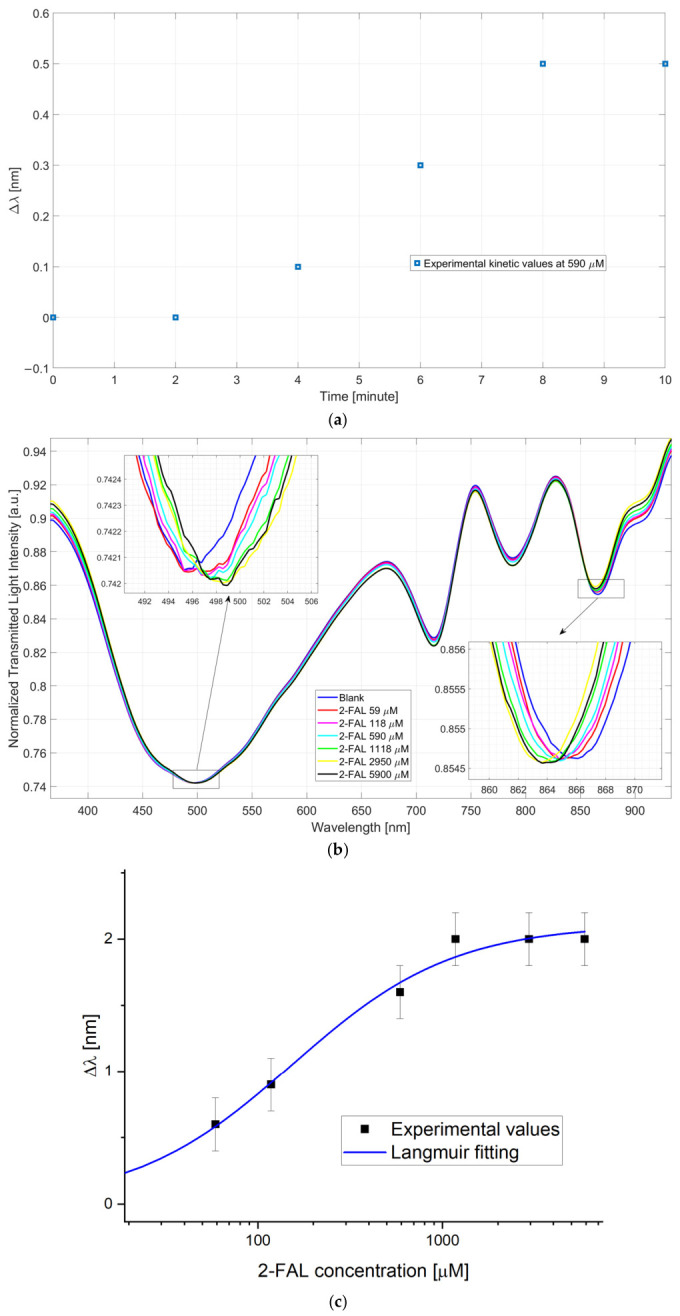
Characterization of the conventional D-shaped POF covered by MIP-GNRs sensors: (**a**) MIP-analyte binding kinetic acquired in a time interval of 10 min total every 2 min at a fixed 2-FAL concentration of 590 µM solution in ultrapure water: variation of the resonance wavelength as a function of time. (**b**) LSPR spectra obtained by normalizing the transmitted spectrum acquired by the sensor chip with respect to that of the reference chip. The inserts show magnification of the indicated resonance wavelengths. (**c**) LSPR wavelength variation (with respect to the blank) as a function of 2-FAL concentration for the resonance around 490 nm.

Figure 6a reports the kinetics of MIP-analyte binding, showing the variation in the resonance wavelength over incubation time at a fixed 2-FAL concentration of 590 μM. LSPR spectra, obtained by normalizing the transmitted spectrum acquired by the sensor chip to that of the reference chip, were acquired every 2 min for 10 min. As reported in Figure 6a, after 8 min of incubation, a stable signal was obtained (t_1/2_ = 5.4 min). Therefore, a 10 min incubation time was used for all binding tests.

Figure 6b shows the LSPR spectra obtained from the sensor’s transmitted spectra normalized to the reference spectrum, for different 2-FAL concentrations ranging from 59 µM to 5900 µM. As shown in Figure 6b, two distinct and exploitable resonance features were present, as shown in the figure inserts.

The LSPR spectra obtained with the conventional D-shaped POF covered by MIP-GNRs configuration show two minima at approximately 490 nm and 860 nm (see Figure 6b), similar to [22]. Among these, the first resonance (around 490 nm) demonstrated higher sensitivity, as reported in [22]. In particular, as 2-FAL concentrations increase, a progressive shift toward longer wavelengths (red-shift) in the LSPR response was observed. Figure 6c shows the dose–response curve obtained by using Equation (1) to fit the experimental data derived from monitoring the first resonance (around 490 nm). In particular, the resonance wavelength shift (Δλ), calculated with respect to the blank (ultrapure water without analyte), is reported on the *y*-axis as a function of the 2-FAL concentration in ultrapure water (*x*-axis).

Three different sensors were fabricated using the same procedure described in Section 3.1 and tested according to the protocol reported in Section 2.7 to evaluate the reproducibility of the fabrication process and the sensing response. The error bar shown in Figure 6c was equal to 0.2 nm and corresponded to the maximum standard deviation obtained from measurements performed on the three sensors (*n* = 3) under the same experimental conditions.

Table 1 reports the Langmuir fitting parameters related to the “Conventional D-shaped POF covered by MIP-GNRs”, while Table 2 summarizes the corresponding chemical parameters.

### 4.3. Trench in POF Filled by MIP-GNRs: Dose–Response Curve for 2-FAL Detection in Ultrapure Water

**Figure 7:**
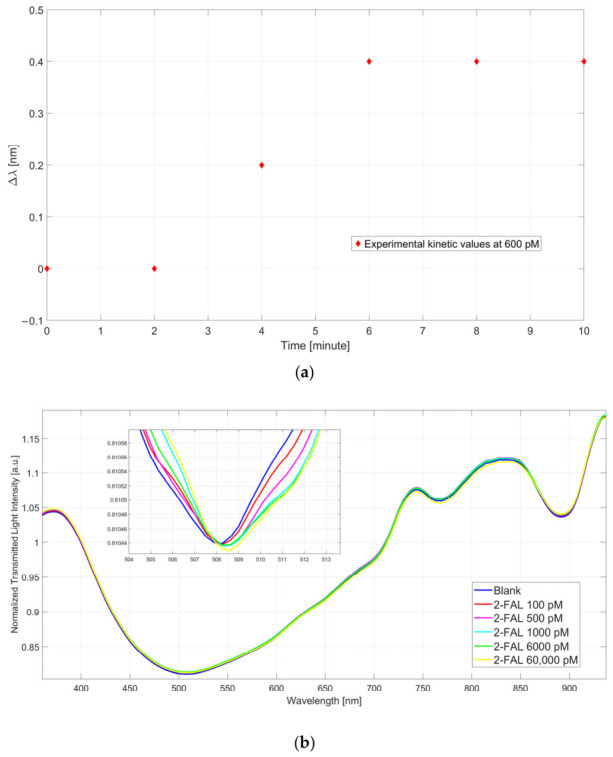
Characterization of the trench in POF filled by MIP-GNRs sensors: (**a**) MIP-analyte binding kinetics acquired during a time interval (10 min total) every 2 min in a fixed 2-FAL 600 pM solution in ultrapure water: variation of the resonance wavelength as a function of time. (**b**) LSPR spectra obtained by normalizing the transmitted spectrum acquired by the sensor chip with respect to that of the reference chip, for different concentrations of 2-FAL from 100 pM to 60,000 pM. The insert shows a zoom of the indicated resonance wavelengths.

Figure 7a reports the kinetics of MIP-analyte binding, i.e., the resonance wavelength variation as a function of incubation time at a fixed 2-FAL concentration of 600 pM. LSPR spectra, obtained by normalizing the transmitted spectrum acquired by the sensor chip to that of the reference chip, were acquired every 2 min for 10 min. As reported in Figure 7a, a stable signal was observed after 6 min of incubation (t_1/2_ = 4 min), and an incubation time of 10 min can be used for all binding tests.

Figure 7b shows the normalized spectra of the “Trench in POF filled by MIP-GNRs” sensor configuration, tested at different 2-FAL concentrations ranging from 100 pM to 60,000 pM, following the protocol described in Section 2.7 and the experimental measurement setup reported in Section 2.6.

As shown in Figure 7b, increasing the 2-FAL concentration results in a progressive shift in the LSPR response, with the resonance wavelengths moving toward higher values (red-shift), in agreement with the resonance response around 490 nm of the “Conventional D-shaped POF covered by MIP-GNRs” sensor previously presented.

The resonance wavelengths were determined by applying a window to the functions around the resonance regions and smoothing them a second time (smoothing factor of 10).

**Figure 8:**
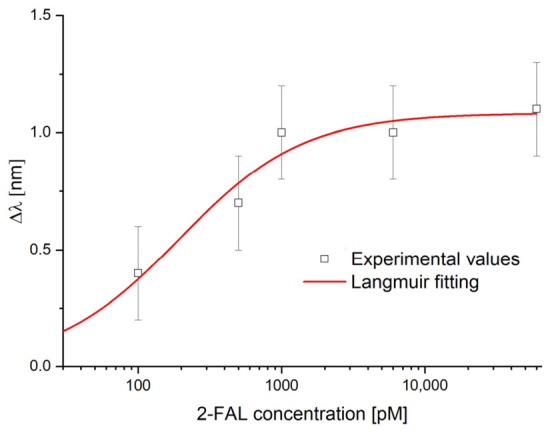
LSPR wavelength variation (with respect to the blank) as a function of 2-FAL concentration.

Figure 8 shows the dose–response curve obtained by applying Equation 1 to fit the experimental data derived from monitoring the LSPR response. In particular, the resonance wavelength shift (Δλ), calculated with respect to the blank (ultrapure water without analyte), is plotted on the *y*-axis as a function of the 2-FAL concentration in ultrapure water on the *x*-axis.

The error bar in Figure 8 is equal to 0.2 nm and is calculated as explained in Section 2.7. The Langmuir fitting parameters are shown in Table 3, while Table 4 summarizes the corresponding analytical parameters obtained as described in Section 2.7.

### 4.4. Selectivity Tests

To test the selectivity of the proposed “Trench in POF filled by MIP-GNRs” sensor configuration, several tests were performed.

#### 4.4.1. Test on Trench in POF Filled by NIP-GNRs

Selectivity tests were initially conducted using the same probe filled with the NIP. In this case, the absence of recognition cavities should allow only weak and nonspecific interactions between the analyte and the polymer. To this end, 2-FAL solutions in the range of 100 pM–60,000 pM were tested, similarly to the MIP-based configuration, following the same protocol (Section 2.7) and using the same experimental setup (Figure 1b).

**Figure 9:**
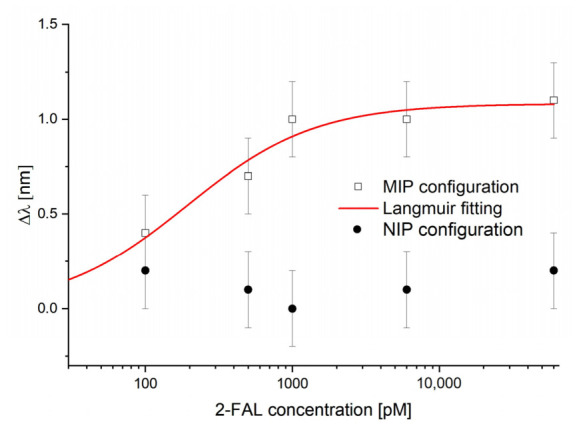
Comparison of the “Trench in POF filled by MIP-GNRs” and the “Trench in POF filled by NIP-GNRs” response for the detection of 2-FAL in pure water.

Figure 9 shows a comparison of the dose–response curves for the MIP and NIP configurations. As shown, the NIP’s LSPR wavelength remains unchanged as the 2-FAL concentration increases, confirming the sensor’s selectivity due to recognition cavities in the MIP.

#### 4.4.2. Test on Trench in POF Filled by MIP-GNRs with Other Possible Interferents

An additional test was then conducted to evaluate the sensor’s response in the presence of potential interfering substances. In particular, 5-hydroxymethylfurfural (5-HMF), a furanic compound with a molecular structure similar to that of 2-FAL, which is also formed in milk and dairy products, often at higher concentrations than 2-FAL, and atrazine (ATZ), which has a completely different molecular structure from furanic compounds but may interfere through non-specific interactions with the MIP.

**Figure 10:**
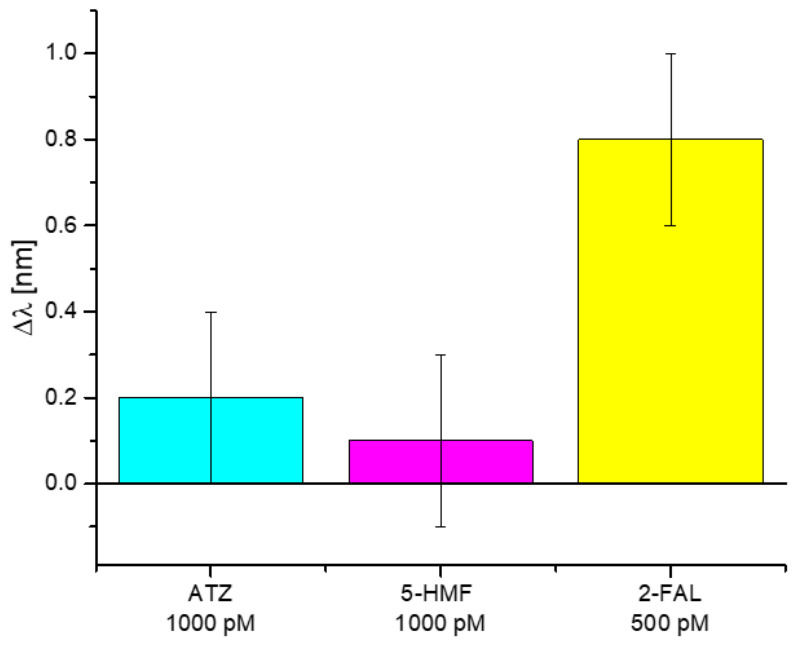
Comparison between the LSPR wavelength variation registered in aqueous solutions of 1000 pM ATZ, 1000 pM 5-HMF, and 500 pM 2-FAL.

Figure 10 shows that both 5-HMF and ATZ solutions at a concentration of 1000 pM produce no statistically significant variation in the LSPR response compared to that obtained for the 2-FAL solution at a concentration of 500 pM.

### 4.5. Tests on Real Matrix

The “Trench in POF filled by MIP-GNRs” sensor was also used to detect 2-FAL concentration in a real matrix. Specifically, a commercially available liquid infant milk (Section 2.5) was tested at various dilution ratios. Specifically, given that the concentration range of 2-FAL in this type of matrix varies from 0.87 μM to 2.36 μM [34], two dilutions of the real sample were performed in order to obtain concentration levels within the sensor’s operating range. Dilution ratios of 1:10,000 (label A) and 1:4000 (label B) in ultrapure water were considered to verify whether the concentration range suitable for the determination was actually reached after the dilutions. The test was performed following the same measurement protocol described above (Section 2.7).

**Figure 11:**
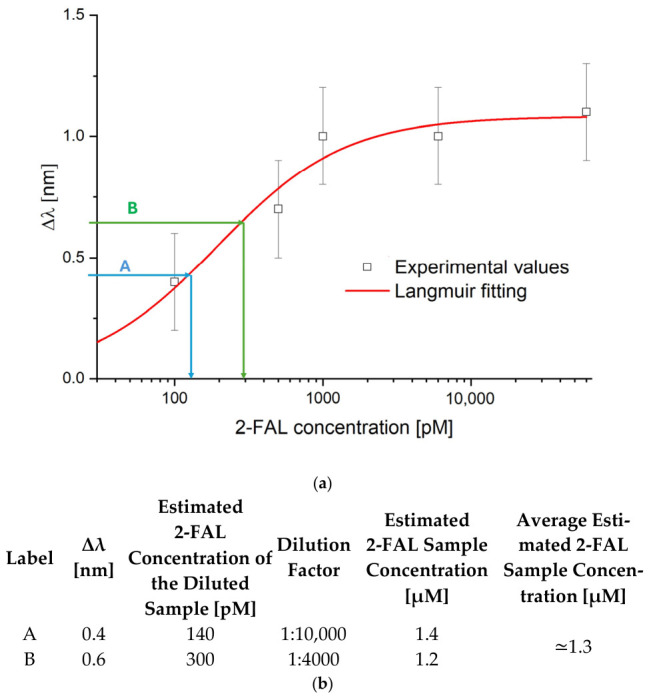
Response of the “Trench in POF filled by MIP-GNRs” setup to liquid infant formula samples tested at different dilution factors and estimated 2-FAL concentration detected. (**a**) Estimated 2-FAL concentration of two samples diluted in ultrapure water: A (diluted 1:10,000) and B (diluted 1:4000) using the dose–response curve obtained in ultrapure water (Figure 7b). (**b**) Table summarizing the wavelength shifts (Δλ) obtained for diluted samples A and B, the estimated 2-FAL concentrations of the diluted samples, and the actual undiluted sample.

The LSPR wavelength shifts (relative to the blank solution) for the two diluted samples can be used to estimate the concentration of 2-FAL in the real sample, together with the Langmuir fit to the dose–response curve (Figure 8). More specifically, in Figure 11a, each LSPR wavelength shift value plotted on the *y*-axis (obtained at different dilutions) can be used to graphically determine the corresponding 2-FAL concentration on the *x*-axis. Obviously, the Langmuir relationship can also be used to estimate concentration, provided the parameters are known.

Figure 11b shows a table that summarizes the wavelength variations (Δλ) obtained at different dilution factors, the estimated concentration of 2-FAL in the diluted samples derived from the Langmuir fit, and the estimated concentration of 2-FAL in the original sample.

The concentration of 2-FAL in the milk sample can be estimated by multiplying the concentration read on the *x*-axis of Figure 11a for the diluted samples by the respective dilution factor (as summarized in Figure 11b). From the experimental values A and B reported in Figure 11b, the concentration of 2-FAL in the analyzed milk sample is estimated at approximately 1.3 μM, a value consistent with those reported in the literature [34].

### 4.6. Comparative Analysis

To provide a more comprehensive comparison between the two sensor configurations investigated here, additional measurements were carried out outside the concentration ranges reported in Figure 6b and Figure 7b.

In particular, the “Conventional D-shaped POF coated with MIP-GNRs” configuration was tested with 2-FAL at low concentrations ranging from 59 pM to 5.9 µM (increasing the concentration in ten-fold steps), and the same curve points shown in Figure 6b were then repeated.

Moreover, the highly sensitive sensor configuration (“Trench in POF filled by MIP-GNRs”) has been tested at 2-FAL high concentrations (from 590 nM to 5900 μM, those used to test “Conventional D-shaped POF coated with MIP-GNRs”), after the 2-FAL low concentration range (the same concentration reported in Figure 7b).

**Figure 12:**
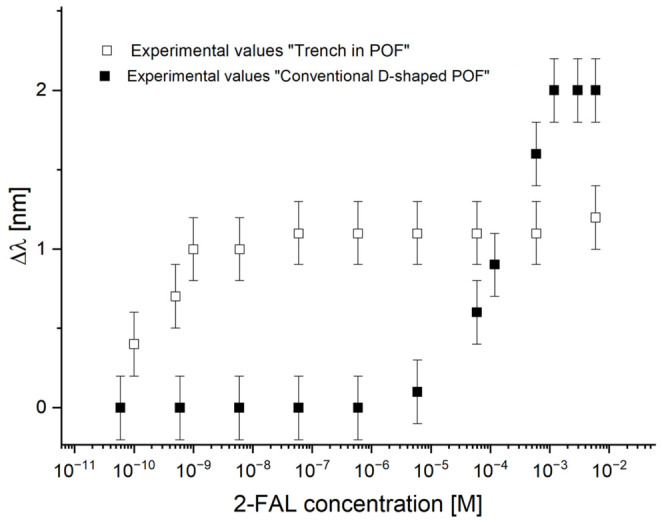
LSPR wavelength variation (with respect to the blank) as a function of 2-FAL concentration ranging from 59 pM to 5900 µM, for both sensor configurations.

Figure 12 summarizes the complete set of experimental points obtained for both sensing configurations over the extended concentration ranges.

The results further highlight a significant difference in sensing performance between the two optical–chemical sensors.

In this work, the interaction between the receptor and the analyte exploits the well-known characteristics of MIPs, in particular the presence of multiple selective binding sites with different affinity constants. As discussed in previous studies, the sensitivity of the optical transducer determines its ability to monitor one or more selective binding sites within the MIP [30,35].

Furthermore, in the proposed “Trench in POF filled by MIP-GNRs” configuration, not only is the optical transduction mechanism different from that of the “Conventional” D-shaped POF coated with an MIP-GNRs layer, but also the morphology of the MIP receptor (and its sites) and, consequently, their affinity for the template. More specifically, in the “Conventional” D-shaped POF coated with MIP-GNRs configuration, the MIP receptor is a thin layer placed on the exposed POF core; whereas, in the trench-based sensor, the receptor is a “MIP mass” embedded in the optical waveguide core.

## 5. Discussion

Several LSPR-based sensors have been reported in the literature, coupling different types of plasmonic nanostructures with an MIP designed to selectively recognize a specific target molecule.

For example, glass-based substrates have been extensively studied: researchers in [36] used gold nanoparticles (AuNPs) coupled with a polydopamine-based MIP for the detection of enrofloxacin (ENRO), achieving a LOD of 0.17 µM, while [37] employed silver nanoparticles (AgNPs) in an acrylamide-based MIP to detect tetrabromobisphenol-A (TBBPA) with a LOD of 8.6 nM. Similarly, LSPR sensors have been integrated onto silica optical fibers; for example, a LOD of 0.112 nM for ascorbic acid using AgNPs was achieved in [38].

More sophisticated fabrication techniques, such as [39], in which an LSPR sensor is realized on a glass substrate with gold nanodisk arrays patterned via colloidal lithography. While this approach enables well-defined plasmonic nanostructures, it involves complex, costly fabrication processes that require specialized equipment and expertise. The sensor response was tested by increasing the pentagalloyl glucose (PGG) concentration, measuring LSPR peak shifts with a spectrometer under continuous-flow conditions. Although the device exhibited a clear dose–response behavior in the range 1–160 μM, no explicit LOD was reported.

In [22], in the context of POFs, two LSPR-based sensor configurations were presented: a conventional D-shaped plastic POF and a tapered POF, both coupled with an MIP for TNT detection, embedding GNS. The experimental setup consisted of a simple transmission configuration using a halogen lamp and a spectrometer, and measurements were performed by dropping the analyte solution onto the sensing region. The conventional configuration exhibited a LOD of 2.4 µM, whereas the tapered POF configuration achieved a significantly improved LOD of 0.72 µM.

In the present study, the “Conventional D-shaped POF covered by MIP-GNRs” configuration, which differs from [22] primarily in the target molecule (2-FAL) and the use of Nanorods (GNRs) instead of nanostars, yielded a LOD of 11.29 µM, comparable to that reported in [22] for TNT, 2.4 µM.

Finally, by machining a micro-trench directly into the POF core and filling it with the MIP-GNR mixture, the sensitive layer is transformed from a simple external coating into an integral part of the waveguide core. This configuration, “Trench in POF filled by MIP-GNRs”, significantly improved binding sensitivity, achieving a LOD of 27 pM, corresponding to an approximately six-order-of-magnitude enhancement over the conventional D-shaped POF configuration. The same holds for a previously investigated sensor for 2-FAL, based on a micro-trench configuration with the same MIP [23], but without LSPR. In that sensor, the LOD was as low as 34 nM, which is three orders of magnitude higher than that obtained here. This significant improvement demonstrates that integrating MIPs as optical waveguides into the light-propagation path, together with suitable gold nanoparticles, increases coupling sensitivity compared to excitation via evanescent waves in surface-coated fibers.

The fact that MIPs obtained by the bulk polymerization method proposed here may contain sites with highly different affinities for the template is well known [35]. The interaction with sites of higher affinity, which are usually formed at lower concentrations in the polymer, is active at lower template concentrations and requires a more sensitive transduction method.

Table 5 summarizes the LSPR-MIP-based sensing configurations, highlighting the superior sensitivity of the trench-filled approach proposed in this work. In particular, Table 5 reports, for each sensor configuration, the type of metal nanostructures employed, the analyte used in the binding test, and the LOD obtained.

Regarding sensors’ stability, it must first be noted that MIP-based receptors offer significant advantages over biological receptor layers, including industrial scalability, low cost, and better resistance to harsh environments, resulting in longer shelf life, etc. [40,41]. More specifically, regarding the MIP receptor used in this work, long-term stability, as well as storage conditions and shelf life, depend on the polymer’s intrinsic characteristics; in fact, it is well known that MIPs have high stability [22,35].

It is worth noting that the optical response is based on the variation in resonance wavelength (Δλ), not the resonance wavelength itself (λ); therefore, the sensor’s reproducibility is very good batch-to-batch and after the regeneration phase. The reusability was also verified in previous works and here as well, showing that the MIP’s sites can be regenerated at least five times without losing sensor performance [42].

## 6. Conclusions

In this work, two different plasmonic sensing configurations based on modified POFs and MIPs with gold nanorods for the selective detection of furfural were developed and characterized. One configuration is the proposed optical–chemical sensor, whereas the second is a conventional POF-based configuration used for comparison. In particular, the conventional configuration is based on a D-shaped POF with the exposed core coated with a thin MIP-GNRs layer, demonstrating a LOD in the micromolar range (11 µM), consistent with the literature on POF-MIP-LSPR sensors. The proposed sensor in this research is the “MIP-GNR-filled POF trench” configuration, in which integrating the MIP directly into the fiber core turns the MIP-GNRs from an outer coating (conventional approach) into an active optical waveguide. As demonstrated by experimental results, the proposed sensor configuration led to a significant improvement over the conventional D-shaped configuration, achieving an LOD of 27 pM instead of 11.29 µM, an increase of six orders of magnitude. The proposed sensing system also demonstrated excellent selectivity toward 2-furfural in the presence of interfering molecules with similar chemical structures, confirming the successful development of MIP-specific recognition sites. Furthermore, the sensor’s applicability was successfully validated by detecting 2-FAL in a real-world matrix; an infant milk solution was tested, yielding results consistent with those obtained in water solutions. The results obtained in this real-world scenario were consistent with those in water solutions, confirming the sensor’s robustness and its ability to perform well even in complex biological or food environments. The proposed sensing system offers a cost-effective, portable, and highly sensitive solution for environmental, food, and industrial monitoring, paving the way for the detection of other harmful contaminants at ultra-trace levels using simple equipment and low-cost POF-based chips.

## Figures and Tables

**Table 1:** The Langmuir fitting parameters relative to the “Conventional D-shaped POF covered by MIP-GNRs” configuration.

Δλ_0_ [nm]		Δλ_max_ [nm]		K [µM]		Statistics	
Value	St. Err.	Value	St. Err.	Value	St. Err.	Reduced χ	R^2^
0.0025	0.079	2.11	0.06	155	26	0.18	0.99

**Table 2:** Analytical parameters of the conventional D-shaped POF covered by MIP-GNRs.

S_low c_ [nm/µM]	LOD [µM]	*K*_aff_ [(µM)^−1^]
0.014	11	0.006 ± 0.0001

**Table 3:** The Langmuir fitting parameters relative to “Trench in POF filled by MIP-GNRs”.

Δλ_0_ [nm]		Δλ_max_ [nm]		K [pM]		Statistics	
Value	St. Err.	Value	St. Err.	Value	St. Err.	Reduced χ	Adj. R-Square
0.0025	0.077	1.08	0.06	192	63	0.16	0.97

**Table 4:** Chemical parameters of the “Trench in POF filled by MIP-GNRs”.

S_low c_ [nm/pM]	LOD [pM]	*K*_aff_ [(pM)^−1^]
0.0056	27	0.005 ± 0.002

**Table 5:** Summary of the LSPR-based sensor configurations combined with MIP coupled to plasmonic nanostructures.

Sensor Configuration	Nanostructure Type	Analyte	LOD	Ref.
Glass substrate coupled with MIP and AuNPs	Gold nanoparticles (AuNPs)	Enrofloxacin (ENRO)	0.17 µM (61.1 ng/mL with a molecular weight of 359.4 g/mol)	[36]
Glass substrate coupled with MIP and AgNPs	Silver nanoparticles (AgNPs)	Tetrabromobisphenol A (TBBPA)	8.6 nM (4.68 ng/mL with a molecular weight of 543.9 g/mol)	[37]
Silica optical fiber core coupled with MIP and AgNPs	Silver nanoparticles (AgNPs)	Ascorbic Acid (AA)	0.112 nM	[38]
Glass substrate patterned with gold nanodisk coupled with MIP	Gold nanodisks	Pentagalloyl glucose (PGG)	<1 µM	[39]
Conventional D-shaped POF covered by MIP-GNSs	Gold nanostars (GNSs)	TNT	2.4 µM	[22]
Tapered POF covered by MIP-GNSs	Gold nanostars (GNSs)	TNT	0.72 µM	[22]
Conventional D-shaped POF covered by MIP-GNRs	Gold Nanorods (GNRs)	2-FAL	11 µM	This work
Trench in POF filled by MIP-GNRs	Gold Nanorods (GNRs)	2-FAL	27 pM	This work

## Data Availability

The original contributions presented in this study are included in the article. Further inquiries can be directed to the corresponding authors.

## Abbreviations

The following abbreviations are used in this manuscript:

POF	Plastic Optical Fiber
SPR	Surface Plasmon Resonance
LSPR	Localized Surface Plasmon Resonance
MIP	Molecularly Imprinted Polymer
NIP	Non-Imprinted Polymer
GNRs	Gold Nanorods
PMMA	Polymethyl Methacrylate
RI	Refractive Index
LOD	Limit of Detection
2-FAL	2-Furaldehyde
MAA	Methacrylic Acid
AIBN	2,2′-Azobisisobutyronitrile
